# Prescription dose and fractionation predict improved survival after stereotactic radiotherapy for brainstem metastases

**DOI:** 10.1186/1748-717X-7-107

**Published:** 2012-07-11

**Authors:** Jonathan E Leeman, David A Clump, Rodney E Wegner, Dwight E Heron, Steven A Burton, Arlan H Mintz

**Affiliations:** 1 Department of Radiation Oncology, University of Pittsburgh Cancer Institute, 5230 Centre Avenue, Pittsburgh, PA, 15232, USA; 2 Department of Neurological Surgery, University of Pittsburgh Cancer Institute, 200 Lothrop Street, Suite 400, Pittsburgh, PA, 15213, USA; 3 Department of Otolaryngology, Head & Neck Surgery, University of Pittsburgh Cancer Institute, 200 Lothrop Street, Suite 200, Pittsburgh, PA, 15213, USA

**Keywords:** Stereotactic radiosurgery, Brain metastases, Brainstem, Fractionation

## Abstract

**Background:**

Brainstem metastases represent an uncommon clinical presentation that is associated with a poor prognosis. Treatment options are limited given the unacceptable risks associated with surgical resection in this location. However, without local control, symptoms including progressive cranial nerve dysfunction are frequently observed. The objective of this study was to determine the outcomes associated with linear accelerator-based stereotactic radiotherapy or radiosurgery (SRT/SRS) of brainstem metastases.

**Methods:**

We retrospectively reviewed 38 tumors in 36 patients treated with SRT/SRS between February 2003 and December 2011. Treatment was delivered with the Cyberknife™ or Trilogy™ radiosurgical systems. The median age of patients was 62 (range: 28–89). Primary pathologies included 14 lung, 7 breast, 4 colon and 11 others. Sixteen patients (44%) had received whole brain radiation therapy (WBRT) prior to SRT/SRS; ten had received prior SRT/SRS at a different site (28%). The median tumor volume was 0.94 cm^3^ (range: 0.01-4.2) with a median prescription dose of 17 Gy (range: 12–24) delivered in 1–5 fractions.

**Results:**

Median follow-up for the cohort was 3.2 months (range: 0.4-20.6). Nineteen patients (52%) had an MRI follow-up available for review. Of these, one patient experienced local failure corresponding to an actuarial 6-month local control of 93%. Fifteen of the patients with available follow-up imaging (79%) experienced intracranial failure outside of the treatment volume. The median time to distant intracranial failure was 2.1 months. Six of the 15 patients with distant intracranial failure (40%) had received previous WBRT. The actuarial overall survival rates at 6- and 12-months were 27% and 8%, respectively. Predictors of survival included Graded Prognostic Assessment (GPA) score, greater number of treatment fractions, and higher prescription dose. Three patients experienced acute treatment-related toxicity consisting of nausea (n = 1) and headaches (n = 2) that resolved with a short-course of dexamethasone.

**Conclusion:**

SRT/SRS for brainstem metastases is safe and achieves a high rate of local control. We found higher GPA as well as greater number of treatment fractions and higher prescription dose to be correlated with improved overall survival. Despite this approach, prognosis remains poor and distant intracranial control remains an issue, even in patients previously treated with WBRT.

## Background

Brain metastases develop in 20-40% of cancer patients representing the most common manifestation of intracranial malignancy [1]. These lesions can result in devastating clinical consequences, particularly when they involve brainstem structures. Options for management of brainstem metastases include whole brain radiation therapy (WBRT) or SRT/SRS [2-4]. Surgical access, however, is often limited in cases of brainstem metastases. In regards to WBRT, there are concerns regarding cognitive effects as well as the potential for durable local control in radioresistant tumors such as renal cell or melanoma metastases. The safety of SRT/SRS for brainstem metastases remains an important question given the proximity to critical structures and potential for treatment-related toxicity [5]. Studies assessing the safety and efficacy of SRT/SRS for metastases to the brainstem have been accumulating from different institutions over the past decade [6-15]. In this study, we analyze our institution’s experience using linac-based SRT/SRS for the treatment of brainstem metastases to determine the safety and efficacy of this treatment approach. 

**Table 1:** Characteristics of patients with brainstem metastases

**Characteristic**	**Value**
**Patients (F/M), n**	36, (17/19)
**Lesions, n**	38
**Median age (range), y**	62 (28-89)
**Primary Malignancy, n (%)**	
Lung	14 (39%)
Breast	7 (19%)
Colon	4 (11%)
Other	11 (31%)
**Symptoms, n (%)**	25 (39%)
Headache	8 (22%)
Weakness	6 (17%)
Ataxia	7 (19%)
Visual Changes	6 (17%)
**Median KPS score (range)**	80 (60-90)
**Median GPA score (range)**	1.5 (0-3.5)
**RPA class, n (%)**	
I	4 (11%)
II	30 (83%)
III	2 (6%)
**Number of intracranial metastases at time of SRS, n (%)**	
1	9 (24%)
2 to 4	23 (61%)
>4	6 (16%)
**Median interval between primary diagnosis and SRS (range), mo**	16 (1-190)
**Median tumor volume (cc), range**	0.94 (0.01-4.2)
**Location of treated brainstem metastasis, n (%)**	
Midbrain	11 (29%)
Pons	25 (66%)
Medulla	2 (5%)
**WBRT for brain metastases before SRS, n (%)**	18 (47%)
**Median SRS dose (range), Gy**	17 (12-24)
**Treatment Fractions, n (%)**	
1	20 (56%)
2	2 (6%)
3	13 (36%)
5	1 (3%)
**Treatment Modality, n (%)**	
Cyberknife	34 (89%)
Trilogy	4 (11%)

## Methods

### Patient population

Patient characteristics are presented in Table 1. We retrospectively analyzed the outcomes of 36 patients with 38 brainstem metastases who received SRT/SRS treatment between February 2003 and December 2011 at the University of Pittsburgh Cancer Institute. The median age was 62 (range: 28–89), seventeen patients were male and 19 patients were female. Of the lesions treated, 25 were located in the pons, 11 in the midbrain, and 2 in the medulla. Primary pathologies included 14 lung, 7 breast, 4 colon and 11 others. Sixteen patients (44%) had received WBRT prior to SRT/SRS; ten (28%) had received prior SRT/SRS to a different site. At the time of their SRT/SRS consult, 25 patients (69%) were symptomatic with neurological complaints including weakness, headaches and ataxia. Furthermore, 29 of the patients (76%) had other distant brain metastases at the time of their consult. Karnofsky Performance Status (KPS), Graded Prognostic Assessment (GPA) [16] and Recursive Partitioning Analysis (RPA) [17] were determined for all of the patients.

### Simulation and planning

Each patient was comfortably positioned on the computerized tomography (CT) simulation table and a custom relocatable thermoplastic mask was fabricated. A thin-slice high resolution CT with intravenous contrast was then obtained while the patient was immobilized. The acquired images were then transferred to the treatment planning workstation and fused with pre-treatment thin-slice (1.2 mm) contrast enhanced spoiled gradient recalled acquisition in steady state (SPGR) sequence magnetic resonance imaging (MRI) utilizing commercially available fusion software. The tumor volume and any surrounding critical structures were manually delineated by a radiosurgical team inclusive of a radiation oncologist, a medical physicist, and a neurosurgeon. The planning target volume was defined as the contrast-enhancing tumor with no margin. Dose volume histograms were calculated for the target volume and nearby critical structures and were utilized to select the optimal treatment plan. An ideal SRT/SRS plan provided coverage of at least 95% of the prescription dose to the PTV while sparing surrounding organs at risk. If surrounding organs at risk were deemed to be at excess risk for toxicity, a plan with lower PTV coverage was accepted. Radiosurgery was performed using CyberKnife^TM^ Robotic Radiosurgery System (Accuray, Inc., Sunnyvale, CA) for 34 lesions and Trilogy^TM^ Radiosurgery System (Varian Medical Systems, Palo Alto, CA) for 4 lesions, one of which was treated in 3 fractions.

### Follow-up

Follow-up neurologic examination and MRI (or CT scanning if ineligible for MRI) were performed at 2 months after SRT/SRS, every 2–3 months for the 1st year, and at 3 to 6 monthly intervals thereafter. Imaging was performed to assess changes in tumor size, to identify the development of any new tumors, and to evaluate the risk of peri-tumoral reactive swelling. A significant change in tumor size was defined as either an increase or decrease of 2 mm in the contrast enhancing dimensions in any single plane of the tumor in accordance with our institutional definition of treatment response. Distant failure was defined as the development of new brain metastases outside the original SRT/SRS treatment volume. Of the patients who had available follow-up data, the presence of extracranial progression was also assessed.

### Statistics

Survival time was computed from the commencement of SRT/SRS. Survival curves and median survival were calculated using the Kaplan–Meier method. Factors affecting survival from the time of brain metastasis diagnosis were determined using the Cox proportional hazards model. All statistical tests were carried out using SPSS Version 15.0 (SPSS, Chicago, IL). The project was reviewed and approved by the University of Pittsburgh Institutional Review Board.

## Results

Thirty-eight tumors in 36 patients were treated. The median tumor volume was 0.94 cm^3^ (range: 0.01-4.2) with a median prescription dose of 17 Gy (range: 12–24) delivered in 1–5 fractions prescribed to the 80% isodose line. The median minimum tumor dose was 15.6 Gy and the median maximum tumor dose was 20.25 Gy. Of the 36 patients, 20 were treated with a single fraction (56%), 2 in two fractions (6%), 13 in 3 fractions (36%) and 1 in 5 fractions (3%). Dose selection and fractionation were based on various factors including tumor volume, location, timing and total dose of prior radiation therapy.

### Local control

**Figure 1:**
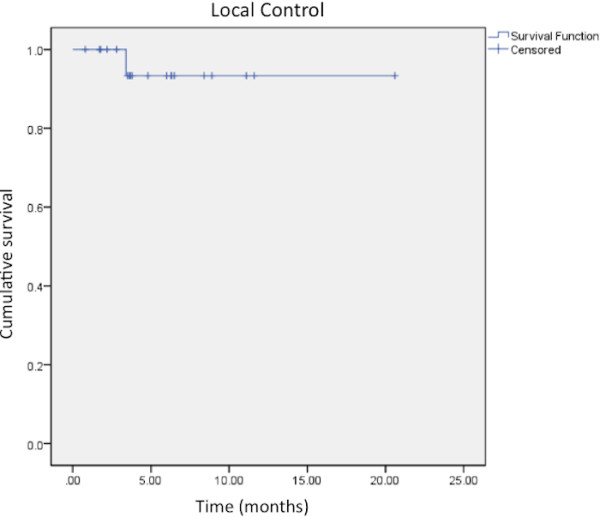
**Kaplan-Meier analysis of local control after SRT/SRS treatment of a brainstem metastasis.** The 6 month and 12 month actuarial control rates were 93%.

Median follow-up from time of SRT/SRS for the cohort was 3.2 months (range: 0.4-20.6). Nineteen patients (52%) had an MRI follow-up available for review. Of these, one patient experienced local failure corresponding to an actuarial 6-month local control of 93% (Figure 1). With only one local failure, significant predictors of local control could not be ascertained.

### Intracranial control

**Figure 2:**
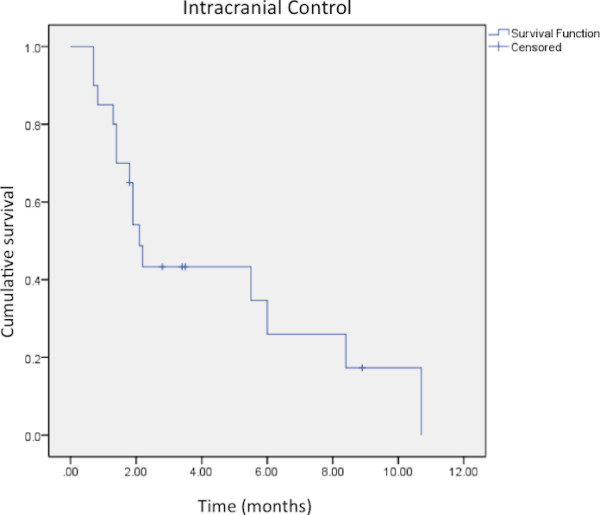
**Kaplan-Meier analysis of intracranial control after SRT/SRS treatment of a brainstem metastasis.** The median time to distant intracranial failure was 2.1 months.

Fifteen of the patients with available follow-up imaging (79%) experienced intracranial failure outside of the treatment volume. Of these fifteen patients, six (40%) had undergone prior WBRT, although WBRT was not found to be significant predictor of intracranial control (p = 0.09) The median time to distant intracranial failure was 2.1 months with a 6-month intracranial control rate of 21% (Figure 2).

### Overall survival

**Figure 3:**
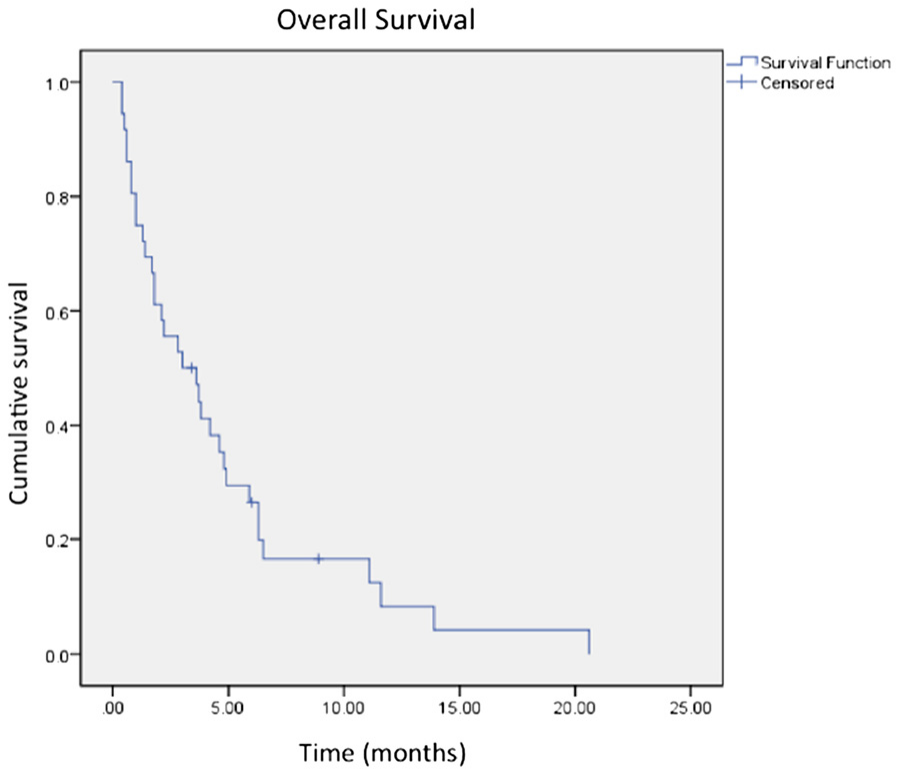
**Kaplan-Meier analysis of overall survival after SRT/SRS treatment of a brainstem metastasis.** Median overall survival was found to be 3 months.

The actuarial overall survival rates at 6- and 12-months were 27% and 8%, respectively (Figure 3). Predictors of survival included Graded Prognostic Assessment (GPA) score, number of treatment fractions, and higher prescription dose. GPA was found to predict overall survival (p = 0.009) as patients with a GPA score of 0–1 had a median survival of 2.1 months and patients with a score of 1.5-2.5 had a median survival of 4.2 months. There were only 3 patients with GPA ≥ 3 with a median survival of 1 month, partly because one patient had a rapid decline in his neurological condition and went to hospice after receiving only 2 out of 3 prescribed fractions. RPA was not found to be a predictor of overall survival as 30/36 patients were RPA class II. Number of fractions was also found to be a significant predictor of overall survival (p = 0.01); patients treated with a single fraction had a median survival of 2.2 months while patients treated in 3 fractions had a median survival of 4.8 months. Similarly, prescription dose was found to be a predictor of overall survival (p = 0.01); patients treated with a prescription dose of <20 Gy had a median survival of 2.1 months while those treated with ≥ 20 Gy survived a median of 4.8 months. Of the twenty patients with known cause of death, 8 were determined to have died from causes related to central nervous system (CNS) dysfunction while 12 died from extracranial causes.

### Toxicity

Three patients experienced acute treatment-related toxicity consisting of nausea (n = 1) and headaches (n = 2) that resolved with a short-course of dexamethasone. No grade 3 or higher toxicities were observed.

## Discussion

In this study, we have assessed the efficacy of linac-based SRT/SRS for the treatment of metastases located in the brainstem and determined rates of local control, intracranial control and overall survival. Furthermore, we have identified factors that are associated with improved overall survival within this subset of patients that may be useful for stratification of patients with brainstem metastases who are being considered as candidates for SRT/SRS.

Prior studies examining SRS for brainstem metastases are summarized in Table 2. In 1999, Huang et al. [8] first assessed SRS for brainstem metastases using Gamma Knife technology (Elekta Medical System, Stockholm, Sweden). With a median prescription dose of 16 Gy and a mean tumor volume of 1.1 mL, they found a local control rate of 95% and a median survival of 9 months with a 27% rate of adverse events. Only active extracranial disease was found to be a predictor of poor survival. Koyfman et al. [11] again used Gamma Knife with a median prescribed dose of 15 Gy for brainstem metastases and found a median survival of 5.8 months with a local control rate of 85% with a 12% complication rate. Lower KPS, larger tumor volume, lower GPA and lower Score Index for Radiosurgery in Brain Metastases (SIR) [18] were all associated with shorter survival. Using linac-based SRS with a median prescribed dose of 15 Gy, Hatiboglu et al. [7] found a median survival of 4.2 months with a local control rate of 76%. They determined that larger tumor volume and male sex were associated with poorer survival. Their rate of adverse events was 20%.

Our data further refine the findings of other authors as well as provide new and important considerations in optimizing outcomes. The data demonstrate that SRT/SRS for brainstem metastases provides effective local control (93%) with failure documented in only one out 19 lesions with available follow-up imaging. This is consistent with the reports mentioned above and findings from other institutions that demonstrate local control rates of 92-100% [6,8,9,12,15] after SRS treatment of a brainstem metastasis. Others have published local control rates that have been slightly lower, ranging from 76-85% [7,10,11,13,14]. This variation is potentially attributable to differences in the radiographic definition of local control, which is not often reported.

We found the median overall survival of patients treated with SRT/SRS for brainstem metastases to be 3 months, somewhat less than others who have reported median survival in the range of 9–12 months after SRS for brainstem metastases [6,8,10,14]. The poor survival in our cohort may be the result of advanced and progressive extracranial disease at the time of presentation; the median GPA score was 1.5 and only 4 patients were classified as RPA class I. Furthermore, of the 20 patients with a known cause of death, 12 (60%) died due to systemic causes that were not related to brain metastases indicating that the burden of extracranial disease in our cohort was relatively high. Importantly, we do not require that patients have controlled extracranial disease to qualify for SRT/SRS treatment. This difference may account for much of the discrepancy in overall survival observed between our study and reported findings. Other studies of SRS for brainstem metastases have demonstrated a relationship between active extracranial disease and shorter survival [8,14].

We found higher GPA score to be correlated positively with increased survival as has also been demonstrated by Koyfman et al. [11]. Specifically, patients with a GPA score of 1.5-2.5 survived twice as long as patients with a score of 0–1. We did not have sufficient patients with GPA > 2.5 for adequate assessment above this threshold, again pointing to the advanced nature of disease in our patient pool. We were unable to assess RPA as a prognostic factor as too few of our patients were classified as RPA I or RPA III. Identification of factors associated with prolonged survival is critical for selection of appropriate treatment candidates, especially given the cost of radiosurgical techniques and potential for toxicity.

A new finding not previously reported is the observation that a greater number of fractions and higher prescription dose were also found to be positively correlated with overall survival. Patients treated with ≥20 Gy in 3 fractions survived significantly longer than patients treated with <20 Gy in 1 fraction. This is likely due to the fact that patients with smaller lesions or advanced extracranial disease are preferentially treated with single fraction regimens whereas patients with larger lesions or well-controlled systemic disease receive fractionated regimens. Lorenzoni et al. [15] found a similar correlation between higher prescription dose and prolonged survival. Additionally, Vogelbaum et al. [19] have demonstrated that with the use of the RTOG 90–05 dosing scheme, smaller tumors treated with 24 Gy were better controlled than larger tumors treated with a smaller dose.

Despite concerns surrounding radiosurgical treatment of the brainstem, our rate of acute adverse effects was quite low (8%). Furthermore, we found no high grade toxicity or new neurological deficits, only headache and nausea that resolved with a short course of steroids. This is in keeping with several other studies that have demonstrated complication rates in the range of 0-10% [6,9,12-15]. The median follow-up time in this study was just 3.2 months due to the poor survival of these patients. As such, while we found low rates of toxicity, it remains possible that patient survival was too short for late toxicities to manifest. These findings are encouraging as they indicate that SRT/SRS for brainstem metastases is a rather safe treatment despite the theoretical potential for serious side effects.

The majority of studies have assessed the use of Gamma Knife for management of brainstem metastases, Our study represents the third report, to our knowledge, examining the use of linac-based platforms for treatment of these lesions. As Table 2 demonstrates, rates of local control, survival and adverse effects are comparable between studies using Gamma Knife or linac platforms suggesting that linac systems may be utilized safely and effectively for treatment of brainstem metastases.

**Table 2:** Prior studies examining SRS for brainstem metastases

**Study**	**Treatment modality**	**Patients, n**	**Mean age, y**	**Median tumor volume, mL**	**Median prescribed SRS Dose, Gy**	**Median survival, mo**	**Local tumor control, %**	**Factors associated With shorter survival**	**Complication rate**
Huang et al. [8]	Gamma Knife	26	56	1.1	16	9	95	Presence of active extracranial disease	27%
Shuto et al. [13]	Gamma Knife	25	57.1	2.1 (mean)	13 (mean)	4.9	77	N/A	8%
Fuentes et al. [6]	Gamma Knife	28	57.7	2.1 (mean)	19.6 (mean)	12	92	N/A	0%
Yen et al. [14]	Gamma Knife	53	57.3	2.8 (mean)	17.6 (mean)	11	87	Presence of extracranial disease	0%
Hussain et al. [9]	Gamma Knife	22	60 (median)	0.9	16	8.5	100	N/A	5%
Kased et al. [10]	Gamma Knife	42	55 (median)	0.26	16	9	85	Multiple metastases, melanoma primary	10%
Lorenzoni et al. [15]	Gamma Knife	25	54	0.6 (mean)	20 (mean)	11.1	95	KPS <80, uncontrolled primary tumor, radiotherapy, SRS < 18 Gy	0%
Koyfman et al. [11]	Gamma Knife	43	59 (median)	0.37	15	5.8	85	Lower KPS, larger tumor volume, SIR, GPA	12%
Hatiboglu et al. [7]	Linac-based SRT/SRS	60	61 (median)	1	15	4.2	76	Tumor volume ≥4 mL, male sex	20%
Lin et al. [12]	Linac-based SRT/SRS	45	59.9	0.4	14	11.6	91	Lower KPS	4%
Present Study	Linac-based SRT/SRS	36	61	0.94	17	3	93	Lower GPA, lower prescription dose, fewer fractions	8%

Interestingly, our study found that treatment with higher dose predicts improved survival but we were unable to make any claim regarding local control as only a single local failure was detected in our cohort. As such, it remains possible that improved local control resulting from higher overall dose is contributing to longer survival but our study was not sufficiently powered to make this conclusion. The low rates of toxicities associated with this therapy suggest that fractionation of therapy may appropriately mitigate adverse effects and allow for safer treatment with higher dosing which may additionally contribute to improved rates of survival. In particular patients with smaller lesions and well controlled extracranial disease may benefit most from the use of higher dosing (≥20 Gy). Fractionation may be considered for larger brain metastases given radiobiological advantages as well as safety concerns for sensitive adjacent structures.

In light of our findings, we do not believe that conservative management with medical therapy alone is recommended for patients with brainstem metastases as SRT/SRS confers a benefit in both local control and survival. Furthermore, we have demonstrated that the risks associated with SRT/SRS in patients with poor prognosis are minimal due to the accuracy and conformal nature of the treatment as well as the fact that shorter survival may not allow late toxicities to manifest. Indeed, distant intracranial failure remains an issue, as 47% of our patients had already received prior WBRT and 76% of patients had multiple brain metastases again highlighting the advanced nature of the presenting disease. Unfortunately, given the retrospective nature of our study, we can only speculate on the potential survival of this cohort were they to be managed conservatively without SRT/SRS. Still, with the favorable risk-benefit profile, we believe that SRT/SRS is appropriately indicated in patients with brainstem metastases and advanced intracranial and/or extracranial disease.

Other limitations of our study include our sample number which was somewhat small, preventing certain comparisons due to power limitations. Additionally, while only 52% of patients in our study had follow-up imaging available for review, this is likely due, in part, to the poor prognosis in this particular group of patients. The number of studies examining SRT/SRS for brainstem metastases is steadily increasing. A meta-analysis would more definitively determine the efficacy and safety of SRT/SRS in this setting and more clearly define optimal treatment regimens and prognostic factors.

## Conclusions

SRT/SRS for brainstem metastases is safe and achieves a high rate of local control; therefore, often evading progressive disease and neurologic death. Patients treated with higher prescription dose and a fractionated regimen demonstrate increased survival. GPA score may be an appropriate prognostic measure of estimated survival that has now been corroborated by multiple reports. Despite this approach, prognosis remains poor and distant intracranial control remains an issue, even in patients who were previously treated with WBRT.

## Abbreviations

SRT:Stereotactic radiotherapy; SRS:Stereotactic radiosurgery; WBRT:Whole brain radiation therapy; GPA:Graded prognostic assessment; RPA:Recursive partitioning analysis; KPS:Karnofsky performance status; MRI:Magnetic resonance imaging; SPGR:Spoiled gradient recalled acquisition; CT:Computerized tomography; CNS:Central nervous system.

## Competing interests

The authors declare that they have no competing interests.

## Authors contributions

JL participated in data collection and organization as well as preparation of the manuscript, DC also participated in data collection, study design and revision of the manuscript. RW performed statistical analyses and revised the manuscript. DH oversaw the study, its design and its conception and revised the manuscript. SB performed data collection and revised the manuscript. AM also performed data collection and revised the manuscript. All authors read and approved the final manuscript.
